# Preliminary Structure-Activity Relationship on Theonellasterol, a New Chemotype of FXR Antagonist, from the Marine Sponge *Theonella swinhoei*

**DOI:** 10.3390/md10112448

**Published:** 2012-11-05

**Authors:** Valentina Sepe, Raffaella Ummarino, Maria Valeria D’Auria, Orazio Taglialatela-Scafati, Simona De Marino, Claudio D’Amore, Barbara Renga, Maria Giovanna Chini, Giuseppe Bifulco, Yoichi Nakao, Nobuhiro Fusetani, Stefano Fiorucci, Angela Zampella

**Affiliations:** 1 Department of Chemistry of Natural Products, University of Naples “Federico II”, via D. Montesano 49, 80131 Naples, Italy; Email: valentina.sepe@unina.it (V.S.); raffaella.ummarino@unina.it (R.U.); madauria@unina.it (M.V.D.A.); scatagli@unina.it (O.T.-S.); sidemari@unina.it (S.D.M.); 2 Department of Clinical and Experimental Medicine Clinica e Sperimentale, University of Perugia, Nuova Facolta di Medicina e Chirurgia, via Gerardo Dottori 1, S. Andrea delle Fratte, 06132 Perugia, Italy; Email: claudiodamore1983@gmail.com (C.D.A.); barbara.renga@unipg.it (B.R.); fiorucci@unipg.it (S.F.); 3 Department of Pharmaceutical and Biomedical Sciences, University of Salerno, via Ponte don Melillo, 84084 Fisciano (SA), Italy; Email: mchini@unisa.it (M.G.C.); bifulco@unisa.it (G.B.); 4 Department of Chemistry and Biochemistry, Waseda University, 3-4-1 Okubo, Shinjuku-ku, Tokyo, 169-8555, Japan; Email: ayocha@waseda.jp (Y.N.); anobu@fish.hokudai.ac.jp (N.F.)

**Keywords:** marine sponges, *Theonella swinhoei*, steroids, theonellasterol, nuclear receptors, farnesoid-X-receptor, chemical modification, structure-activity relationship

## Abstract

Using theonellasterol as a novel FXR antagonist hit, we prepared a series of semi-synthetic derivatives in order to gain insight into the structural requirements for exhibiting antagonistic activity. These derivatives are characterized by modification at the exocyclic carbon-carbon double bond at C-4 and at the hydroxyl group at C-3 and were prepared from theonellasterol using simple reactions. Pharmacological investigation showed that the introduction of a hydroxyl group at C-4 as well as the oxidation at C-3 with or without concomitant modification at the exomethylene functionality preserve the ability of theonellasterol to inhibit FXR transactivation caused by CDCA. Docking analysis showed that the placement of these molecules in the FXR-LBD is well stabilized when on ring A functional groups, able to form hydrogen bonds and π interactions, are present.

## 1. Introduction

**Figure 1:**
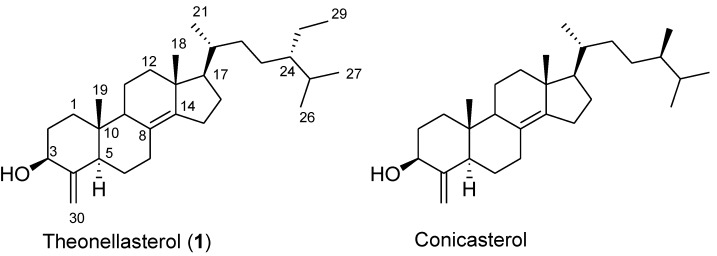
Theonellasterol (**1**) and conicasterol, the parent 4-methylenesteroids from *Theonella* sponges.

Among marine sponges, certainly the *Theonella* species have been proven to be an extraordinary source of unusual new chemical entities, mainly peptides and macrolides, often endowed with impressive biological activities and therefore being promising lead compounds. Additionally the steroidal composition is peculiar in the *Theonella* sponges, which contain the rare 4-methylenesteroids as exclusive components of this biogenetic class. Thus, theonellasterol (**1**) and conicasterol (Figure 1), first isolated in 1981, are considered ideal biomarkers of *Theonella swinhoei* and *Theonella conica*, respectively [1]. These molecules share the same tetracyclic core with the unusual 4-methylene functionality and the rare ∆^8,14^ double bond but differ in the side chain with a 24*S*-ethyl group in theonellasterol and a 24*R*-methyl group in conicasterol. Recently we had the opportunity to analyze the extracts of several *Theonella* collections affording the isolation of anti-inflammatory peptides [2,3,4,5,6,7] and sulfated steroids [8,9,10,11], cytotoxic macrolides [12] and a large number of 4-methylenesteroids demonstrating, for the first time, their ability to target the farnesoid-X-receptor (FXR) and the pregnane-X-receptor (PXR) [13,14,15,16,17,18]. These are two nuclear receptors involved in regulating bile acid synthesis as well as in detoxification and excretion in the liver and gastro-intestinal tract [19,20,21,22] and therefore important pharmacological targets in the treatment of cholestatic disorders [23,24,25]. Cholestasis, a liver disease, represents the main biochemical feature of primary biliary cirrhosis [26,27] (PBC) and sclerosing cholangitis (PSC), two immune-mediated disorders characterized by progressive bile duct destruction for which medical therapy is still insufficiently effective and where investigations are ongoing to identify novel therapeutic approaches [24,25].

Within the family of 4-methylenesteroids from *Theonella swinhoei*, we have identified theonellasterol (**1**) as the first example of a sponge derived highly selective FXR antagonist [28,29,30] demonstrating its pharmacological potential in the treatment of cholestasis. Indeed theonellasterol directly inhibits FXR transactivation caused by CDCA (chenodeoxycholic acid) and reverses the effect of CDCA on the expression of canonical FXR target genes. In rodent models of cholestasis, theonellasterol (**1**) attenuates liver injury caused by bile duct ligation as measured by assessing serum alanine aminostrasferase levels and the extent of liver necrosis at histopathology [28]. 

In this experimental setting we found that the inhibition of FXR reverses the antagonism that this nuclear receptor exerts on basolateral transporters, specifically on MRP-4, thus allowing bile acids secretion from the basolateral membrane of hepatocytes. Indeed, activation of this “alternative” route for bile acids secretion in the presence of bile ducts obstruction, helps bile acids detoxification by the liver. Because extensive bile ducts destruction is the main pathological feature of advanced primary sclerosing cholangitis and primary biliary cirrhosis, identification of FXR antagonists could be beneficial in the treatment of these “orphan” diseases. In addition to cholestasis, FXR antagonism might have pharmacological and clinical relevance in several human disorders, including cancers of the esophagus, stomach and pancreas which express high levels of FXR and whose proliferation is driven by bile acids in a FXR-dependent manner [18].

By docking calculations we have rationalized the binding mode of theonellasterol (**1**) in FXR [28]. Besides the *trans* junction between A/B rings and the unsaturation between C-8 and C-14 cause a different spatial arrangement with respect to the agonist 6-ethylchenodeoxycholic acid (6-ECDCA), a potent synthetic FXR agonist [31], theonellasterol (**1**) competes with 6-ECDCA in occupying FXR ligand binding domain (LBD). This thereby establishes several hydrophobic interactions of its tetracyclic core with aminoacids of the Helices 2–3, 5–7, and 10/11 and notably crucial interaction of the OH at C-3 position and the key aminoacids of LBD (namely Tyr358 in Helix 7, His444 in Helix 10/11, and Trp466 in Helix12) [32].

## 2. Results and Discussion

Theonellasterol **(1**) is the major component of the steroidal fraction of *Theonella swinhoei* and can be isolated in high amounts following a very simple procedure. The availability of reasonable amounts of **1**, its stability, and the presence in the tetracyclic core of functional groups that could be modified, appeared to provide a good opportunity to investigate the effect of chemical transformations on biological activity and to perform the first structure-activity relationship (SAR) study on this new chemotype of FXR antagonist.

As the highly hindered 8,14 double bond was found to be chemically unreactive toward most chemical reagents, there remain two points for chemical modification in the structure of theonellasterol: The exocyclic carbon-carbon double bond at C-4 and the hydroxyl group at C-3. These functionalities were subjected to simple chemical reactions and the products obtained (**2**–**12**) were fully characterized by means of MS, and 1D and 2D NMR spectroscopy. 

**Scheme 1:**
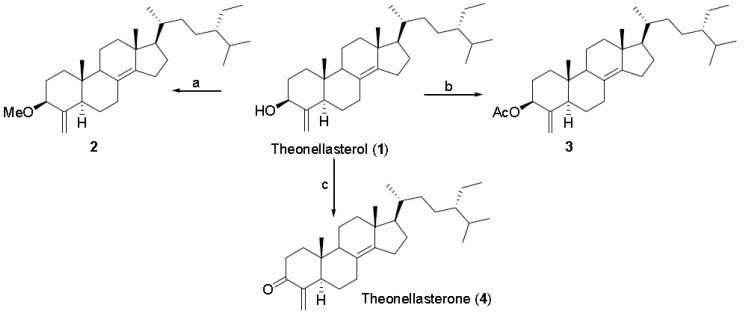
Modification at C-3 hydroxyl group. Reagents and conditions: (**a**) NaH, THF, MeI, 0 °C, 79%; (**b**) Ac_2_O, pyridine (pyr), room temperature (rt), 96%; (**c**) PCC, CH_2_Cl_2_, quantitative yield.

The methyl ether derivative (**2**), the 3-*O*-acetyl derivative (**3**) and the α,β-unsaturated ketone (**4**), already known as theonellasterone [1], were prepared from theonellasterol (Scheme 1) to explore the pharmacophoric role of the hydroxyl group at C-3 as hydrogen bond donor in the FXR-LBD (Scheme 1).

We then reasoned that the configuration at C-3 could also play a substantial role in accommodating the steroid nucleus in the binding site of FXR. Unfortunately, all attempts to obtain the C-3 epimer of theonellasterol through the reduction (NaBH_4_ or LiAlH_4_) of theonellasterone (**4**) failed, invariably producing theonellasterol. Also inversion at C-3 by treatment of theonellasterol with tosyl chloride followed by potassium acetate in water was unsuccessful only resulting in extensive degradation of the starting material. 

**Scheme 2:**
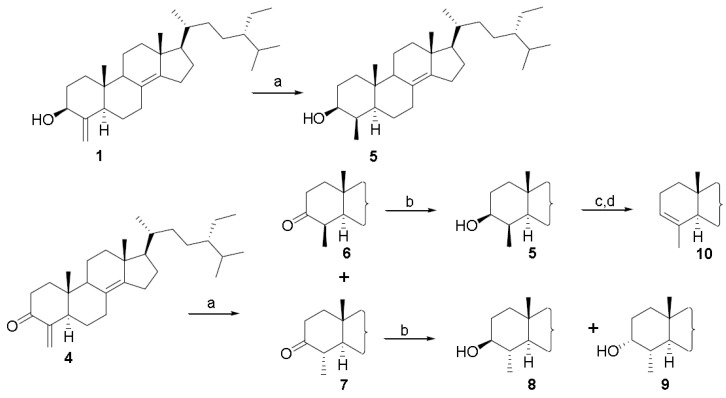
Modification at C-4 double-bond. Reagents and conditions: (**a**) H_2_, 10% Pt/C, THF:MeOH 1:1; (**b**) NaBH_4_, absolute MeOH, 0 °C; (**c**) TsCl, pyr, rt; (**d**) CH_3_COOK, DMF:H_2_O 7:1, reflux, 75%, over two steps.

Next, we turned our attention to reactions of the carbon-carbon double bond on ring A. As judged by the downfield shift of the C-19 methyl proton [1], hydrogenation of theonellasterol on different catalysts (Pt/C, Pd(OH)_2_ Degussa type, PtO_2_) produced exclusively the 4β-methyl derivative (**5**) through the approach of the hydrogen from the α-face of the steroid nucleus (Scheme 2).

To obtain the 4α-methyl theonellasterol derivative, we devised an alternative synthetic procedure via theonellasterone (Scheme 2). 

Theonellasterone (**4**) was reduced to a mixture of the two 4-methyl diasteroisomers (H_2_, 10% Pt/C, THF/MeOH), **6** and **7**, which were efficiently separated by HPLC. In the ^1^H NMR spectrum of **7**, H-4 was observed as a double quartet (2.05, dq, *J* = 14.2, 6.1 Hz) and the large coupling constant with H-5 clearly pointed towards its axial position, thus implying the α-orientation of the methyl group at C-4.

Chemical correlation gave definitive confirmation of the above stereochemical assignment. As depicted in Scheme 2, for the concomitant steric effect played by Me-19 and Me-30, both orientated on the β-face of the steroidal nucleus, NaBH_4_ reduction of **6** afforded exclusively 3β-hydroxy-4β-methyl steroisomer (**5**). On the other hand, reduction of **7** gave a mixture of 3β-hydroxy-4α-methyl theonellasterol derivative (**8**) with its C-3 epimer, 3α-hydroxy-4α-methyl- derivative (**9**).

As previously reported for several natural and synthetic 4-methyl cholestane derivatives [33], in the 4α-methyl-3β-ol derivative (**8**), the 3α-proton resonance is consistently shifted upfield with respect to the corresponding resonances in the 4α-methyl-3α-ol (**9**) and 4β-methyl-3β-ol diasteroisomers (**5**) (*δ*_H_ 2.93 in **8**, *δ*_H_ 3.55 in **9**, *δ*_H_ 3.54 in **5**), thus substantiating the stereochemical assignment reported in Scheme 2. Moreover in the ^1^H NMR of **9**, H-3 was observed as a broad multiplet, allowing its assignment as equatorial and therefore establishing the orientation of the hydroxyl group at C-3 on the α-face of the molecule.

To access the 4β-methyl-3α-ol derivative, the 4β-methyl theonellasterol derivative (**5**) was subjected to a two step sequence involving treatment with tosyl chloride in pyridine followed by potassium acetate in DMF/H_2_O (Scheme 2). Unfortunately the basic treatment of the 3-*O*-tosyl intermediate produced β-elimination with the formation of derivative **10** with the 3,4 double bond. Nevertheless, **10** could be instrumental in the evaluation of the pharmacophoric role played by the oxygen atom on ring A. 

**Scheme 3:**
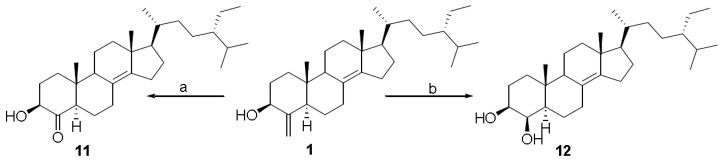
Modification at C-4 double-bond. Reagents and conditions: (**a**) O_3_ solution in CH_2_Cl_2_, −78 °C, then DMS, 84%; (**b**) O_3_ solution in CH_2_Cl_2_, −78 °C, then NaBH_4_, 93%.

Finally we decided to investigate the effects of the introduction of a polar group at C-4 in the binding of theonellasterol (**1**) in FXR-LBD. Oxidative cleavage with ozone (O_3_, CH_2_Cl_2_, −78 °C, 5 min) followed by dimethylsulfide or NaBH_4_ work-up afforded the 4-keto derivative (**11**) and the 4-hydroxy derivative (**12**), respectively (Scheme 3). The presence in the ^13^C NMR spectrum of a signal at *δ*_C_ 212.1 clearly inferred the presence of a ketone at C-4 in **11** that was also confirmed by the chemical shift value of the H-3 resonance, shifted downfield with respect to **5** (*δ*_H_ 3.80 in **11**, *δ*_H_ 3.54 in **5**). In agreement with the steric influence played by Me-19, ^1^H NMR spectrum analysis revealed that the sodium borohydride work-up proceeded in a stereoselective manner affording the exclusive formation of 4β-hydroxy derivative (**12**) as judged by the shape of H-4 as a broad singlet. This is consistent with an equatorial disposition for this proton, and therefore with the axial β-orientation of the hydroxyl group. It was confirmed by the strong downfield shift exhibited by Me-19 (*δ*_H_ 1.12 in **12**, *δ*_H_ 0.63 in **1**) caused by the 1,3-diaxial relationship with the hydroxy group at C-4.

All derivatives of this small library were tested *in vitro*, using a hepatocarcinoma cell line (HepG2 cells) transfected with FXR, RXR, β-galactosidase expression vectors (pSG5FXR; pSG5RXR and pCMV-βgal), and with p(hsp27)TKLUC reporter vector that contains the promoter of the FXR target gene heat shock protein 27 (hsp27) cloned upstream of the Luciferase gene.

**Figure 2:**
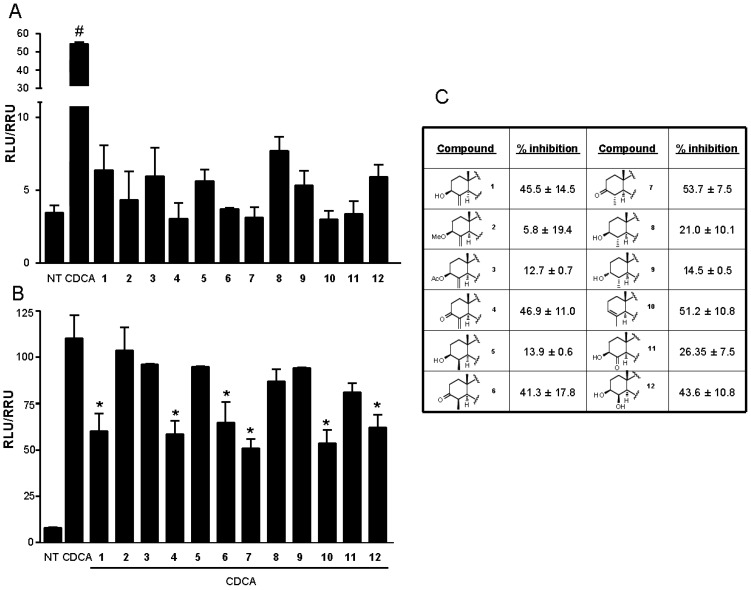
(**A**) Luciferase reporter assay performed in HepG2 transiently transfected with pCMVSPORT-FXR, pSG5-RXR, pGL4.70-Renilla, and p(hsp27)TKLUC vectors and stimulated 18 h with chenodeoxycholic acid (CDCA), 10 μM, and compounds **1**–**12**, 10 μM. ^#^ *P* < 0.05 *vs.* NT (*n* = 4); (**B**) Luciferase reporter assay performed in HepG2 transiently transfected with pCMVSPORT-FXR, pSG5-RXR, pGL4.70-Renilla, and p(hsp27)TKLUC vectors and stimulated 18 h with CDCA, 10 μM, alone or in combination with compounds **1**–**12**, 50 μM. * *P* < 0.05 *vs.* CDCA (*n* = 4); (**C**) Antagonism reported as percent of inhibition normalized to CDCA as 100%.

HepG2 cells were stimulated with compounds **1**–**12** in the presence or in the absence of CDCA (10 µM). As shown in Figure 2A, in accordance with our previously reported data for theonellasterol [28] none of these compounds appears to be an FXR agonist in the transactivation assay.

However, when HepG2 cells transfected with FXR vectors were treated with compounds **1**–**12** (Figure 2B) in the presence of 10 µM CDCA, several derivatives showed inhibitory activity against FXR transactivation induced by CDCA.

**Figure 3:**
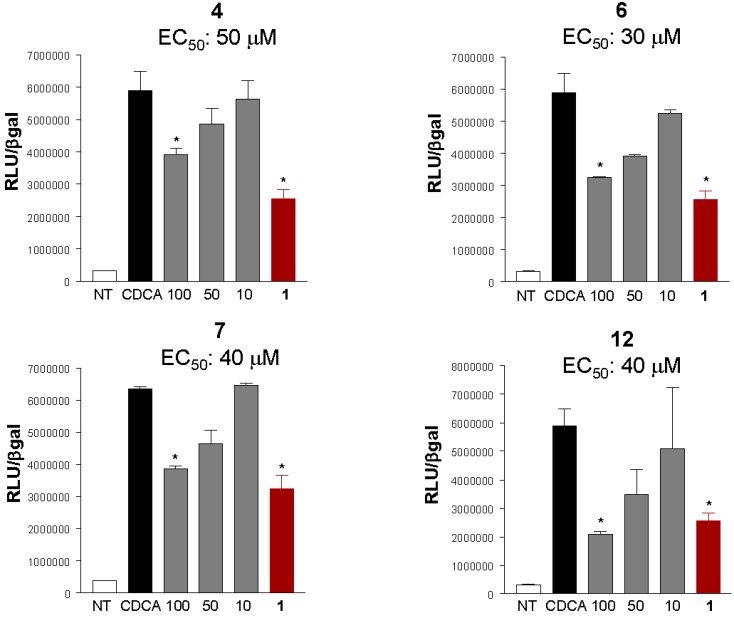
Luciferase reporter assay on HepG2 transiently transfected with pCMVSPORT-FXR, pSG5-RXR, pGL4.70-Renilla, and p(hsp27)TKLUC vectors and stimulated with 10 μM CDCA alone (black bar), or in combination with increasing concentrations (10, 50 and 100 μM, grey bars) of derivatives** 4**, **6**, **7**, **12 **and with theonellasterol (**1**) 100 μM (red bar). ^#^ *P* < 0.05 *vs.* CDCA (*n* = 3).

Out of all the synthetic derivatives obtained in this study, we then selected a subset of compounds (**4**, **6**, **7**, **12**) and their relative efficacy in inhibiting FXR transactivation caused by CDCA was measured in a luciferase reporter assay. Data shown in Figure 3 demonstrated that, in comparison to theonellasterol (**1**) (EC_50_ approximately 50 μM), the selected derivatives had an EC_50_ ranging from 35 to 50 μM. The relative potency in inhibiting FXR transactivation caused by CDCA was similar to that of the parent theonellasterol (**1**) (*i.e.*, 50%–60%).

**Figure 4:**
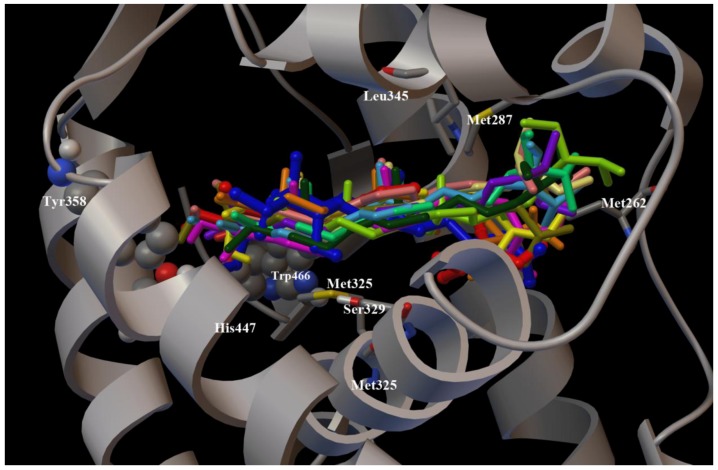
Superimposition of the binding modes of **1**–**12**, and of the co-crystallized agonist 6-ECDCA in the FXR-LBD. The chain A of FXR (pdb code: 1OSV) and the key amino acids (see main text for details) are represented by grey ribbon, cpk and stick and balls respectively colored by atom type (C, grey; O, red; N, dark blue; S, yellow; H, light grey). 6-ECDCA (dark blue), **1** (red), **2** (light yellow), **3** (green), **4** (emerald green), **5** (olive green), **6** (purple), **7** (dark green), **8** (pink), **9** (yellow), **10** (orange), **11** (cyan), and **12** (light pink) are represented by stick and balls.

To clarify the effects of the chemical modifications at C-3 and at C-4 of theonellasterol (**1**) at the atomic level, molecular docking calculations [34] were performed using the X-ray structure of the human FXR-LBD [13,15,17,28,29] (pdb code: 1OSV). In our three dimensional models (Figure 4), all synthetic derivatives **2**–**12** adopt the same positioning in the FXR-LBD with respect to the parent compound theonellasterol (**1**) maintaining hydrophobic contacts of their tetracyclic cores with the receptor (Leu345, Met287, Met325, Met262, Ser329, Trp466), and interacting in a different manner with the key aminoacids Tyr358, His444, and Trp466 (Figure 4) [32].

**Figure 5:**
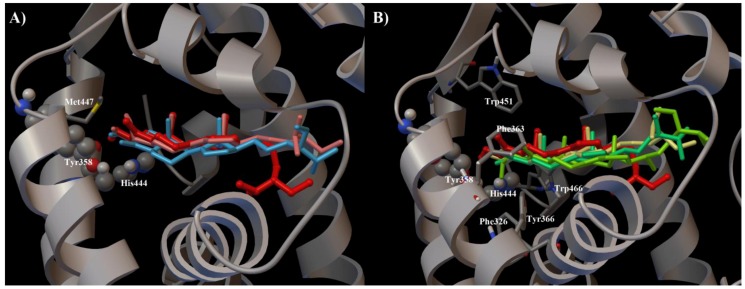
(**A**) Superimposition of the docking poses of **1** (red), **11** (cyan), and **12** (light pink) in the FXR-LBD. (**B**) Three dimensional model of the different interaction pattern of **1** (red), **2** (light yellow), **3** (green), **4** (emerald green), with FXR. In both figures the crucial amino acids (see main text for details) of the receptor are depicted by grey ribbon, cpk, and stick and balls respectively colored by atom type (C, grey; O, red; N, dark blue; S, yellow; H, light grey).

Interestingly, when a hydroxyl group was introduced in place of the exomethylene functionality (Figure 2, panels B and C), the resulting derivative **12** showed an antagonistic activity comparable with **1** thus suggesting a role of hydrogen bond donor for this group in FXR-LBD as confirmed by the inactivity of derivative **11** with a ketone functionality at C-4. In fact, with respect to **1** and **11**, in the derivative **12** the OH group forms an additional hydrogen bond with the side chain of Met447 (Figure 5A). 

Concerning the C-3 hydroxyl group, the complete inefficacy of derivatives **2** and **3** (Figure 2, panels B and C), confirms its role as hydrogen bond donor as demonstrated for **1** (Figure 5B) [28]. 

**Figure 6:**
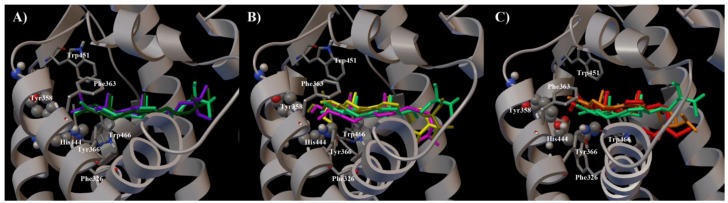
(**A**) Superimposition of the binding modes of **4** (emerald green) with **6** (purple) and **7** (dark green). (**B**) **4** (emerald green) with **5** (olive green), **8** (pink) and **9** (yellow). (**C**) **4** (emerald green) with **1** (red) and **10** (orange) in the FXR-LBD. In all the figures the crucial aminoacids (see main text for details) of the receptor are depicted by grey ribbon, cpk, and stick and balls respectively colored by atom type (C, grey; O, red; N, dark blue; H, light grey).

On the other hand, theonellasterone (**4**) maintained the antagonistic activity, with the ketone functionality at C-3 acting as H bond acceptor through the interactions with the Tyr358 and the His444 as for theonellasterol. As shown in Figure 5B these contacts are hampered in **2** and **3** for the steric effects played by the methyl or acetyl substitution, respectively. Moreover, the binding of theonellasterone (**4**) in the FXR-LBD is also stabilized by additional interactions, mainly a carbonyl-π contact with Tyr358 and His444 and an exomethylene-π interaction with Tyr358 of the aromatic pocket formed by His444, Phe326, Phe363, Tyr358, Tyr366, Trp466 and Trp451 (Figure 5B). These interactions may be also responsible for the retained FXR antagonist activity of **6** and **7** (Figure 6A) with respect to the 4-methyl theonellasterol derivatives (**5**, **8** and **9**), which were found to be inactive towards FXR (Figure 6B). In other words these data suggest that in the 3-keto derivatives the antagonistic activity is also retained when the exomethylene at C-4 is replaced by a methyl group.

Further on, the presence of the above aromatic pocket in the LBD is also responsible for the ability of the derivative **10** to antagonize CDCA in FXR transactivation suggesting that modifications at C-3 and C-4 with concomitant elimination of the hydroxyl and the exomethylene group could produce active compounds if the π interactions with Tyr358, His444, and Trp451 are maintained (Figure 6C). Therefore, the ability to form hydrogen bonds and π interactions with the FXR-LBD seems to represent the main driving force to obtain stable and efficient sterol-receptor complexes.

## 3. Experimental Section

### 3.1. General

Specific rotations were measured on a Jasco P-2000 polarimeter. High-Resolution ESI-MS spectra were performed with a Micromass Q-TOF mass spectrometer. NMR spectra were obtained on Varian Inova 400 and Varian Inova 500 NMR spectrometers (^1^H at 400 and 500 MHz, ^13^C at 100 and 125 MHz, respectively) equipped with a Sun hardware and recorded in C_6_D_6_ (*δ*_H_ = 7.16 and *δ*_C_ = 128.4 ppm). *J* values are in hertz and chemical shifts (*δ*) are reported in ppm and referred to C_6_HD_5_ as internal standards. HPLC was performed using a Waters Model 510 pump equipped with Waters Rheodyne injector and a differential refractometer, model 401.

Reaction progress was monitored via thin-layer chromatography (TLC) on Alugram^®^ silica gel G/UV254 plates. Silica gel MN Kieselgel 60 (70–230 mesh) from Macherey-Nagel Company was used for column chromatography. All chemicals were obtained from Sigma-Aldrich, Inc. Solvents and reagents were used as supplied from commercial sources with the following exceptions. Tetrahydrofuran and dichloromethane were distilled from calcium hydride immediately prior to use.

Methanol was dried from magnesium methoxide as follow. Magnesium turnings (5 g) and iodine (0.5 g) are refluxed in a small (50–100 mL) quantity of methanol until all of the magnesium has reacted. The mixture is diluted (up to 1 L) with reagent grade methanol, refluxed for 2–3 h then distilled under nitrogen. All reactions were carried out under an argon atmosphere using flame-dried glassware. All reactions were carried out under an argon atmosphere using flame-dried glassware.

### 3.2. Sponge Material and Isolation Procedures for Theonellasterol (**1**)

*Theonella swinhoei* sponge (S07102) was collected at a depth of 10–15 m, Kakeroma Island, Kagoshima prefecture (129, 21, 09’E) on June 22, 2007. The sample was immediately frozen after the collection and kept at -20 °C until the extraction. Frozen sponge (1.20 kg wet weight) was extracted with MeOH (3 L × 3 times). The MeOH extract was evaporated and partitioned between H_2_O and CHCl_3_. The aqueous layer was extracted with *n*-BuOH and the so obtained*n*-BuOH was combined with the CHCl_3_ layer. The combined layers were evaporated and subjected to the modified Kupchan procedure to obtain *n*-hexane (6.7 g), CHCl_3_, and 60% MeOH extracts. The hexane extract was chromatographed by silica gel MPLC using a solvent gradient system from CH_2_Cl_2_ to CH_2_Cl_2_:MeOH 1:1. The fraction eluted with CH_2_Cl_2_:MeOH 995:5 (700 mg) was further purified by HPLC on a Nucleodur 100-5 C18 (5 μm; 10 mm internal diameter × 250 mm) with MeOH:H_2_O (998:2) as eluent (flow rate 5 mL/min) to give 650 mg of theonellasterol (*t*_R_ = 19.6 min). The identity of theonellasterol (**1**) was established by comparison of NMR and mass data with those previously reported [1].

### 3.3. Synthetic Procedures

#### 3.3.1. 3β-*O*-Methyl-theonellasterol **2 **

To a solution of theonellasterol (**1**) (10 mg, 0.023 mmol) in dry THF (5 mL) at 0 °C was added NaH (5.5 mg, 0.23 mmol). After 10 min methyl iodide (28.6 μL, 0.46 mmol) was added and the mixture was left to stand at room temperature for 4 h. The mixture was quenched by addition at 0 °C of methanol (2 mL) and then concentrated *in vacuo*. Ethyl acetate and water were added and the separated aqueous phase was extracted with ethyl acetate (3 × 50 mL). The combined organic phases were washed with water, dried (Na_2_SO_4_) and concentrated. Purification by silica gel eluting with CH_2_Cl_2_ gave the methyl ether **2 **as an amorphous solid (8.0 mg, 79%). [*α*]_25_^D^ = +4.0 (*c *0.75, CH_3_OH); selected ^1^H NMR (400 MHz C_6_D_6_): *δ* 5.49 (1H, s, H-30a), 4.77 (1H, s, H-30b), 3.39 (1H, dd, *J * = 4.5, 11.7 Hz, H-3), 3.33 (3H, s, OCH_3_), 1.06 (3H, d, *J * = 6.9 Hz, H_3_-21), 0.94 (3H, t, *J * = 7.0 Hz, H_3_-29), 0.93 (3H, s, H_3_-18), 0.91 (3H, d, *J * = 7.0 Hz, H_3_-26), 0.90 (3H, d, *J * = 7.0 Hz, H_3_-27), 0.67 (3H, s, H_3_-19). ^13^C NMR (100 MHz C_6_D_6_): *δ* 150.7 (C-4), 143.3 (C-14), 126.8 (C-8), 104.5 (C-30), 83.4 (C-3), 57.6 (C-17), 57.4 (-OCH_3_), 50.4 (C-9), 50.1 (C-5), 47.0 (C-24), 43.5 (C-13), 40.8 (C-10), 38.2 (C-12), 37.5 (C-1), 35.7 (C-20), 34.6 (C-22), 31.0 (C-2), 30.2 (C-6), 29.7 (C-25), 27.9 (C-7), 27.0 (C-23), 26.5 (C-16), 25.4 (C-15), 23.8 (C-28), 21.2 (C-11), 20.1 (C-27), 19.9 (C-21), 19.6 (C-26), 18.9 (C-18), 13.7 (C-19), 13.0 (C-29); HRESI MS *m/z* 441.4092 (calcd for C_31_H_53_O 441.4096).

#### 3.3.2. 3β-*O*-Acetyl-theonellasterol **3 **

A mixture of theonellasterol (**1**) (10 mg, 0.023 mmol) and acetic anhydride (55 μL, 0.575 mmol) in dry pyridine (10 mL) was left to stand at room temperature for 8 h. Then the solvent was evaporated and purification by silica gel eluting with CH_2_Cl_2_ gave **3 **as an amorphous solid (10.3 mg, 96%). [*α*]_25_^D^ = +0.5 (*c *2.4, CH_3_OH); selected ^1^H NMR (400 MHz C_6_D_6_): *δ* 5.37 (1H, dd, *J * = 4.7, 11.5 Hz, H-3), 5.15 (1H, s, H-30a), 4.69 (1H, s, H-30b), 1.80 (3H, s, CH_3_CO), 1.05 (3H, d, *J * = 6.6 Hz, H_3_-21), 0.94 (3H, t, *J * = 7.0 Hz, H_3_-29), 0.91 (3H, d, *J * = 6.5 Hz, H_3_-26), 0.91 (3H, s, H_3_-18), 0.90 (3H, d, *J * = 6.5 Hz, H_3_-27), 0.63 (3H, s, H_3_-19). ^13^C NMR (100 MHz C_6_D_6_): *δ* 169.7 (CH_3_CO), 149.1 (C-4), 143.4 (C-14), 126.6 (C-8), 104.4 (C-30), 75.1 (C-3), 57.6 (C-17), 50.0 (C-9), 49.6 (C-5), 46.9 (C-24), 43.5 (C-13), 40.3 (C-10), 38.1 (C-12), 36.9 (C-1), 35.7 (C-20), 34.6 (C-22), 30.4 (C-6), 29.9 (C-2), 29.7 (C-25), 27.9 (C-7), 27.0 (C-23), 26.6 (C-16), 25.2 (C-15), 23.8 (C-28), 21.1 (2C, C-11 and CH_3_CO), 20.1 (C-27), 19.9 (C-21), 19.6 (C-26), 18.8 (C-18), 13.6 (C-19), 13.0 (C-29); HRESI MS *m/z* 469.4098 (calcd for C_32_H_53_O 469.4096).

#### 3.3.3. Theonellasterone** 4**

To the solution of theonellasterol (**1**) (100 mg, 0.23 mmol) in dichloromethane (5 mL) was added pyridinium chlorochromate (99 mg, 0.46 mmol). The reaction mixture was stirred at room temperature for 3 h, and then dichloromethane and water were added. The separated aqueous phase was extracted with dichloromethane (3 × 30 mL). The combined organic phases were washed with water, dried (Na_2_SO_4_) and evaporated to dryness. The brown oily residue was passed through a short column of silica gel (10 g) and eluted with CH_2_Cl_2_ to give **4 (**95 mg, quantitative yield) as an amorphous solid. [*α*]_25_^D^ = +2.2 (*c *0.07, CH_3_OH); selected ^1^H NMR (400 MHz C_6_D_6_): *δ* 6.04 (1H, s, H-30a), 4.86 (1H, s, H-30b), 2.39 (1H, dd, *J * = 2.1, 13.8 Hz, H-2a), 1.06 (3H, d, *J * = 6.5 Hz, H_3_-21), 0.95 (3H, t, *J * = 7.3 Hz, H_3_-29), 0.92 (3H, d, *J * = 6.6 Hz, H_3_-26), 0.90 (3H, s, H_3_-18), 0.90 (3H, d, *J * = 6.6 Hz, H_3_-27), 0.57 (3H, s, H_3_-19); ^13^C NMR (100 MHz C_6_D_6_): *δ* 200.9 (C-3), 150.3 (C-4), 143.7 (C-14), 127.4 (C-8), 118.2 (C-30), 57.6 (C-17), 50.0 (C-9), 49.0 (C-5), 46.9 (C-24), 43.4 (C-13), 40.0 (C-10), 38.0 (C-12), 37.5 (C-1), 35.9 (C-2), 35.7 (C-20), 34.6 (C-22), 34.5 (C-6), 29.8 (C-25), 27.9 (C-7), 27.1 (C-3), 26.6 (C-16), 25.3 (C-15), 23.8 (C-28), 20.9 (C-11), 20.1 (C-27), 19.9 (C-21), 19.6 (C-26), 18.8 (C-18), 12.9 (C-29), 12.8 (C-19). HRESI MS *m/z* 425.3795 (calcd for C_30_H_49_O 425.3783).

#### 3.3.4. (24*S*)-24-Ethyl-4β-methyl-5α-cholestan-3β-ol **5**

An oven-dried 50 mL flask was charged with 10% platinum on carbon (20 mg) and theonellasterol (**1**) (100 mg, 0.23 mmol) and the flask was evacuated and flushed with argon. Absolute methanol (10 mL) and dry THF (10 mL) were added, and the flask was flushed with hydrogen. The reaction was stirred at room temperature under H_2_ for 5 h. The mixture was filtered through Celite, and the recovered filtrate was concentrated to give 85 mg of pure **5 **as an amorphous solid (86%). [*α*]_25_^D^ = +6.7 (*c *0.25, CH_3_OH); selected ^1^H NMR (400 MHz C_6_D_6_): *δ* 3.54 (1H, m, H-3), 1.05 (3H, d, *J * = 6.2 Hz, H_3_-21), 0.95 (3H, d, ovl, H_3_-30), 0.94 (3H, s, H_3_-18), 0.94 (3H, t, ovl, H_3_-29), 0.91 (3H, d, *J* = 7.0 Hz, H_3_-26), 0.89 (3H, d, *J * = 7.0 Hz, H_3_-27), 0.75 (3H, s, H_3_-19). ^13^C NMR (100 MHz C_6_D_6_): *δ* 143.8 (C-14), 127.0 (C-8), 74.1 (C-3), 57.7 (C-17), 51.7 (C-9), 48.6 (C-5), 46.9 (C-24), 43.8 (C-13), 40.9 (C-4), 40.5 (C-10), 38.1 (C-12), 37.9 (C-1), 37.8 (C-2), 35.7 (C-20), 34.6 (C-22), 30.8 (C-6), 29.7 (C-25), 27.9 (C-7), 27.1 (C-23), 26.6 (2C, C-15 and C-16), 23.8 (C-28), 20.1 (2C, C-11 and C-27), 19.9 (C-21), 19.6 (C-26), 19.1 (C-18), 15.5 (C-30), 15.4 (C-19), 12.9 (C-29); HRESI MS *m/z* 429.4076 (calcd for C_30_H_53_O 429.4096).

#### 3.3.5. (24*S*)-24-Ethyl-4β-methyl-5α-cholestan-3-one **6** and (24*S*)-24-Ethyl-4α-methyl-5α-cholestan-3-one **7**

An oven-dried 50 mL flask was charged with 10% platinum on carbon (20 mg) and theonellasterone (100 mg, 0.23 mmol) and the flask was evacuated and flushed with argon. Absolute methanol (10 mL) and dry THF (10 mL) were added, and the flask was flushed with hydrogen. The reaction was stirred at room temperature under H_2_ for 5 h. The mixture was filtered through Celite, and the recovered filtrate was concentrated. The mixture was purified by HPLC on a Nucleodur Isis 100-5 C18 (5 μm; 4.5 mm internal diameter × 250 mm) with MeOH:H_2_O (999.5:0.5) as eluent (flow rate 1 mL/min) to give 39 mg (40% from **5**) of **6** (*t*_R_ = 55 min) and 30 mg (31% from **5**) of **7** (*t*_R_ = 60 min) as amorphous solids.

##### 3.3.5.1. (24*S*)-24-Ethyl-4β-methyl-5α-cholestan-3-one **6**

[*α*]_25_^D^ = −0.7 (*c *0.06, CH_3_OH); selected ^1^H NMR (400 MHz C_6_D_6_): *δ* 2.38 (1H, m, H-4), 1.05 (3H, d, *J * = 6.8 Hz, H_3_-21), 0.95 (3H, t, *J * = 7.0 Hz, H_3_-29), 0.95 (3H, d, *J * = 7.0 Hz, H_3_-30), 0.93 (3H, s, H_3_-18), 0.91 (3H, d, *J * = 7.0 Hz, H_3_-26), 0.90 (3H, d, *J * = 7.0 Hz, H_3_-27), 0.73 (3H, s, H_3_-19). ^13^C NMR (100 MHz C_6_D_6_): *δ* 196.0 (C-3), 143.4 (C-14), 126.6 (C-8), 57.6 (C-17), 50.8 (C-9), 49.6 (C-5), 48.7 (C-4), 46.9 (C-24), 43.4 (C-13), 39.0 (C-10), 38.3 (C-12), 37.9 (C-1), 37.7 (C-2), 35.6 (C-20), 34.6 (C-22), 30.4 (C-6), 29.7 (C-25), 27.8 (C-7), 27.0 (C-23), 26.6 (C-16), 26.5 (C-15), 23.8 (C-28), 20.1 (C-27), 19.9 (C-21), 19.7 (C-11), 19.6 (C-26), 19.0 (C-18), 14.6 (C-19), 14.5 (C-30), 12.9 (C-29); HRESI MS *m/z* 427.3936 (calcd for C_30_H_51_O 427.3940).

##### 3.3.5.2. (24*S*)-24-Ethyl-4α-methyl-5α-cholestan-3-one **7**

[*α*]_25_^D^ = −12.3 (*c *0.10, CH_3_OH); selected ^1^H NMR (400 MHz C_6_D_6_): *δ* 2.05 (1H, dq, *J* = 6.1, 14.2 Hz, H-4), 1.09 (3H, d, *J * = 6.1 Hz, H_3_-30), 1.06 (3H, d, *J * = 6.7 Hz, H_3_-21), 0.94 (3H, s, H_3_-18), 0.94 (3H, t, *J* = 7.4 Hz, H_3_-29), 0.91 (3H, d, *J * = 6.7 Hz, H_3_-26), 0.89 (3H, d, *J * = 6.7 Hz, H_3_-27), 0.70 (3H, s, H_3_-19). ^13^C NMR (100 MHz C_6_D_6_): *δ* 199.1 (C-3), 143.2 (C-14), 126.4 (C-8), 57.5 (C-17), 53.8 (C-9), 49.5 (C-5), 46.9 (C-24), 45.5 (C-4), 43.4 (C-13), 39.0 (C-10), 38.5 (C-12), 38.1 (2C, C-1 and C-2), 35.7 (C-20), 34.6 (C-22), 30.1 (C-6), 29.7 (C-25), 27.9 (C-7), 27.0 (C-23), 26.8 (C-15), 26.6 (C-16), 23.8 (C-28), 20.7 (C-11), 20.1 (C-27), 19.9 (C-21), 19.6 (C-26), 18.9 (C-18), 13.4 (C-30), 12.9 (C-29), 12.3 (C-19); HRESI MS *m/z* 427.3944 (calcd for C_30_H_51_O 427.3940).

#### 3.3.6. (24*S*)-24-Ethyl-4α-methyl-5α-cholestan-3β-ol** 8 **and (24*S*)-24-Ethyl-4α-methyl-5α-cholestan-3α-ol** 9 **

To a solution of **7** (30 mg, 0.070 mmol) in dry methanol (5 mL) was added NaBH_4_ (13 mg, 0.35 mmol) at 0 °C. After 30 min the reaction was quenched by addition of MeOH (3 mL) and then concentrated *in vacuo*. Ethyl acetate and water were added and the separated aqueous phase was extracted with ethyl acetate (3 × 30 mL). The combined organic phases were washed with water, dried (Na_2_SO_4_) and concentrated. The mixture was purified by HPLC on a Nucleodur Isis 100-5 C18 (5 μm; 4.5 mm internal diameter × 250 mm) with MeOH:H_2_O (999.5:0.5) as eluent (flow rate 1 mL/min) to give 15 mg (50% from **7**) of **8** (*t*_R_ = 47.5 min) and 10 mg of **9 **(34% from **7**) (*t*_R_ = 50 min) as amorphous solids.

##### 3.3.6.1. (24*S*)-24-Ethyl-4α-methyl-5α-cholestan-3β-ol** 8**

[*α*]_25_^D^ = −2.1 (*c *0.02, CH_3_OH); selected ^1^H NMR (400 MHz C_6_D_6_): *δ* 2.93 (1H, m, H-3), 2.01 (1H, dt, *J * = 6.0, 12.6 Hz, H-4), 1.06 (3H, d, *J * = 6.5 Hz, H-21), 1.02 (3H, d, *J* = 6.0 Hz, H-30), 0.95 (3H, s, H-18), 0.94 (3H, t, *J * = 7.3 Hz, H-29), 0.91 (3H, d, *J * = 7.3 Hz, H-26), 0.90 (3H, d, *J * = 7.3 Hz, H-27), 0.71 (3H, s, H-19). HRESI MS *m/z* 429.4088 (calcd for C_30_H_53_O 429.4096).

##### 3.3.6.2. (24*S*)-24-Ethyl-4α-methyl-5α-cholestan-3α-ol** 9**

[*α*]_25_^D^ = +4.1 (*c *0.04, CH_3_OH); selected ^1^H NMR (400 MHz C_6_D_6_): *δ* 3.55 (1H, br m, H-3), 1.05 (3H, d, *J* = 6.5 Hz, H_3_-21), 0.95 (3H, d, *J* = 6.5 Hz, H_3_-30), 0.95 (3H, s, H_3_-18), 0.94 (3H, t, *J * = 6.8 Hz, H_3_-29), 0.91 (3H, d, *J * = 7.0 Hz, H_3_-26), 0.89 (3H, d, *J * = 7.0 Hz, H_3_-27), 0.75 (3H, s, H_3_-19). HRESI MS *m/z* 429.4084 (calcd for C_30_H_53_O 429.4096).

#### 3.3.7. (24*S*)-24-Ethyl-4-methyl-5α-cholest-3-ene** 10**

To a solution of **5** (30 mg, 0.070 mmol) in dry pyridine (5 mL), a solution of tosyl chloride (66 mg, 0.35 mmol) in dry pyridine (5 mL) was added. The solution was stirred at room temperature for 2 h and then concentrated *in vacuo*. The precipitate was re-dissolved in CH_2_Cl_2_, washed with NaHCO_3_ saturated solution and water, dried with Na_2_SO_4_, and then evaporated to dryness to give the 3β-tosylate, which was subjected to the next step without any purification. A solution of 3β-tosylate and CH_3_COOK (7.5 mg, 0.077 mmol) dissolved in DMF (3.5 mL) and water (0.5 mL) was refluxed for 2 h. The solution was cooled at room temperature and then ethyl acetate and water were added. The separated aqueous phase was extracted with ethyl acetate (3 × 30 mL). The combined organic phases were washed with water, dried (Na_2_SO_4_) and evaporated to dryness. Purification by HPLC on a Nucleodur Isis 100-5 C18 (5 μm; 4.5 mm internal diameter × 250 mm) with MeOH:H_2_O (999.5:0.5) as eluent (flow rate 1 mL/min) gave **10** (21 mg, 75% over two steps) as an amorphous solid. [*α*]_25_^D^ = +43 (*c *0.01, CH_3_OH); selected ^1^H NMR (400 MHz C_6_D_6_): *δ* 5.44 (1H, br s, H-3), 1.64 (3H, s, H_3_-30), 1.06 (3H, d, *J * = 6.4 Hz, H_3_-21), 0.96 (3H, s, H_3_-18), 0.94 (3H, t, *J* = 7.5 Hz, H_3_-29), 0.91 (3H, d, *J* = 6.8 Hz, H_3_-26), 0.90 (3H, d, *J * = 6.8 Hz, H_3_-27), 0.81 (3H, s, H_3_-19); HRESI MS *m/z* 411.3985 (calcd for C_30_H_51_ 411.3991).

#### 3.3.8. (24*S*)-24-Ethyl-3β-hydroxyl-5α-cholest-4-one** 11**

A stream of O_3_ was bubbled into CH_2_Cl_2_ (5 mL) at −78 °C until a blue-colored solution resulted. A portion of this solution (4 mL) was added to a solution of **1** (10 mg, 0.023 mmol) in CH_2_Cl_2_ kept under argon at −78 °C. After stirring for 1 h, excess of ozone was removed upon bubbling with N_2_ and the solution was treated with excess dimethylsulfide (2 mL). After 5 h, the solution was concentrated *in vacuo* to remove the solvent and the mixture was purified by HPLC on a Nucleodur Isis 100-5 C18 (5 μm; 4.5 mm internal diameter × 250 mm) with MeOH:H_2_O (999.5:0.5) as eluent (flow rate 1 mL/min) to give 8.4 mg (84%) of **11 **(*t*_R_ = 27.5 min) as an amorphous solid. [*α*]_25_^D^ = +3.2 (*c *0.37, CH_3_OH); selected ^1^H NMR (400 MHz C_6_D_6_): *δ* 3.80 (1H, m, H-3), 1.06 (3H, d, *J * = 6.4 Hz, H_3_-21), 0.94 (3H, t, *J* = 7.4 Hz, H_3_-29), 0.91 (3H, d, *J * = 7.0 Hz, H_3_-26), 0.90 (3H, d, *J * = 7.0 Hz, H_3_-27), 0.83 (3H, s, H_3_-18), 0.45 (3H, s, H_3_-19); ^13^C NMR (100 MHz C_6_D_6_): *δ* 212.1 (C-4), 144.3 (C-14), 125.3 (C-8), 75.2 (C-3), 57.6 (C-17), 56.6 (C-9), 49.6 (C-5), 47.0 (C-24), 44.3 (C-10), 43.4 (C-13), 38.0 (C-1), 35.7 (C-20), 35.1 (C-12), 34.5 (C-22), 33.4 (C-2), 29.8 (C-25), 28.8 (C-7), 27.8 (C-15), 27.1 (C-23), 26.6 (C-16), 23.8 (C-28), 21.5 (C-6), 20.9 (C-11), 20.1 (C-27), 19.9 (C-26), 19.6 (C-21), 18.8 (C-18), 14.4 (C-19), 12.9 (C-29); HRESI MS *m/z* 429.3741 (calcd for C_29_H_49_O_2_ 429.3733).

#### 3.3.9. (24*S*)-24-Ethyl-5α-cholestan-3β,4β-diol **12**

A stream of O_3_ was bubbled into CH_2_Cl_2_ (5 mL) at −78°C until a blue-colored solution resulted. A portion of this solution (4 mL) was added to a solution of theonellasterol (10 mg, 0.023 mmol) in CH_2_Cl_2_ kept under argon at −78 °C. After stirring for 1 h, excess O_3_ was removed upon bubbling with N_2_. To the solution was added methanol (2 mL) and then treated with an excess of NaBH_4_. The solution was stirred at room temperature for 3 h and then concentrated *in vacuo*. The precipitate was re-dissolved in ethyl acetate, washed with water, dried with Na_2_SO_4_ and then evaporated to dryness. The mixture was purified by HPLC on a Nucleodur Isis 100-5 C18 (5 μm; 4.5 mm internal diameter × 250 mm) with MeOH:H_2_O (999.5:0.5) as eluent (flow rate 1 mL/min) to give 9.2 mg (93%) of **12 **(*t*_R_ = 31.5 min) as a amorphous solid. [*α*]_25_^D^ = +6.9 (*c *0.5, CH_3_OH); selected ^1^H NMR (400 MHz, C_6_D_6_): *δ* 3.52 (1H, br s, H-4), 3.28 (1H, dd, *J * = 5.2, 10.6 Hz, H-3), 1.12 (3H, s, H-19), 1.05 (3H, d, *J * = 6.8 Hz, H-21), 0.94 (3H, s, H-18), 0.93 (3H, t, *J* = 7.5 Hz, H-29), 0.90 (3H, d, *J * = 7.2 Hz, H-26), 0.89 (3H, d, *J * = 7.2 Hz, H-27); ^13^C NMR (100 MHz C_6_D_6_): *δ* 143.1 (C-14), 127.3 (C-8), 74.8 (C-3), 72.7 (C-4), 57.6 (C-17), 51.0 (C-9), 49.0 (C-5), 46.9 (C-24), 43.6 (C-13), 38.3 (C-10), 38.2 (C-12), 37.2 (C-1), 35.8 (C-20), 34.6 (C-22), 30.6 (C-6), 29.7 (C-25), 27.9 (C-7), 27.0 (C-23), 26.6 (3C, C-2, C-15 and C-16), 23.8 (C-28), 20.1 (C-11), 20.0 (C-27), 19.9 (C-21), 19.6 (C-26), 19.0 (C-18), 15.4 (C-19), 13.0 (C-29); HRESI MS *m/z* 431.3883 (calcd for C_29_H_51_O_2_ 431.3889).

### 3.4. *In Vitro* FXR Transactivations

HepG2 cells were cultured at 37 °C in Minimum Essential Medium with Earl’s salts containing 10% fetal bovine serum (FBS), 1% L-glutamine and 1% penicillin/streptomycin. The transfection experiments were performed using Fugene HD (Promega) according to the manufacturer’s specifications. HepG2 cells were plated in a 12-well plate at 1 × 10^5^ cells/well. Cells were transfected with 150 ng pCMVSPORT-FXR, 150 ng pSG5-RXR, 200 ng pGL4.70-Renilla and with 500 ng of the reporter vector p(hsp27)-TK-LUC containing the FXR response element IR1 cloned from the promoter of heat shock protein 27 (hsp27). At 24 h post-transfection, agonistic activity was measured by treating cells for 18 h with either 10 µM CDCA (positive control) and compounds **1**–**12**; for antagonistic activity, cells were treated for 18 h with the combination CDCA (10 µM) plus compounds **1**–**12** (50 µM). After treatment, cells were lysed in 100 µL diluted reporter lysis buffer (Promega) and 20 µL cellular lysate was assayed for Luciferase and Renilla activity using the Luciferase or Renilla Assay System (Promega). Luminescence was measured using an automated luminometer. Luciferase activities were normalized for transfection efficiencies by dividing the Luciferase relative light units by Renilla relative light units expressed from cells co-transfected with pGL4.70-Renilla.

### 3.5. Computational Details

All docking calculations by Autodock 4.2 software [34] were performed on 4 × AMD Opteron 16 Core at 2.3 GHz, using a grid box size of 94 × 96 × 68 for chain A of FXR (pdb code:1OSV) [32], with spacing of 0.375 Å between the grid points, and centered at 20.689 (*x*), 39.478 353 (*y*), 10.921 (*z*) between the SCH_3_ of Met262 and the OH group of Thr267, covering the active site of the receptor. The Lamarckian genetic algorithm (LGA) was employed for docking experiments, choosing an initial population of 600 randomly placed individuals. The maximum number of energy evaluations and of generations was set up to 5 × 10^6^ and to 6 × 10^6^ respectively. For all the docked structures, all bonds were treated as active torsional bonds except the bonds in cycles, which were considered fixed together with the receptor. Results that differed by <3.5 Å in positional root-mean-square deviation (RMSD) were clustered together and represented by the most favorable free energy of binding. Illustrations of the 3D models were generated with Python software [35] using MGLTools 1.5.6.

## 4. Conclusions

In conclusion we investigated the effect of chemical transformations on the biological activity of theonellasterol (**1**), performing the first preliminary structure-activity relationship (SAR) on this new chemotype of FXR antagonist. The discovery of a preserved FXR antagonistic activity in derivatives **6**, **7**, and **12** having more synthetically accessible function groups on ring A, opens the way to the design and the preparation of new potential leads in the pharmacological treatment of human FXR-mediated diseases. 

## References

[1] Kho E., Imagawa D.K., Rohmer M., Kashman Y., Djerassi C. (1981). Sterols in marine invertebrates. 22. Isolation and structure elucidation of conicasterol and theonellasterol, two new 4-methylene sterols from the Red Sea sponges *Theonella conica *and *Theonella swinhoei*. J. Org. Chem..

[2] Festa C., De Marino S., Sepe V., Monti M.C., Luciano P., D’Auria M.V., Debitus C., Bucci M., Vellecco V., Zampella A. (2009). Perthamides C and D, two new potent anti-inflammatory cyclopeptides from a Solomon Lithistid sponge *Theonella swinhoei*. Tetrahedron.

[3] Margarucci L., Monti M.C., Mencarelli A., Fiorucci S., Riccio R., Zampella A., Casapullo A. (2012). Heat shock proteins as key biological targets of the marine natural cyclopeptide perthamide C. Mol. BioSyst..

[4] Sepe V., D’Auria M.V., Bifulco G., Ummarino R., Zampella A. (2010). Concise synthesis of AHMHA unit in perthamide C. Structural and stereochemical revision of perthamide C. Tetrahedron.

[5] Festa C., De Marino S., Sepe V., D’Auria M.V., Bifulco G., Andres R., Terencio M.C., Paya M., Debitus C., Zampella A. (2011). Perthamides C-F, potent human antipsoriatic cyclopeptides. Tetrahedron.

[6] Festa C., De Marino S., D’Auria M.V., Monti M.C., Bucci M., Vellecco V., Debitus C., Zampella A. (2012). Anti-Inflammatory cyclopeptides from the marine sponge *Theonella swinhoei*. Tetrahedron.

[7] Festa C., De Marino S., Sepe V., D’Auria M.V., Bifulco G., Debitus C., Bucci M., Vellecco V., Zampella A. (2011). Solomonamides A and B, new anti-inflammatory peptides from *Theonella swinhoei*. Org. Lett..

[8] Festa C., De Marino S., D’Auria M.V., Bifulco G., Renga B., Fiorucci S., Petek S., Zampella A. (2011). Solomonsterols A and B from *Theonella swinhoei*. The first example of C-24 and C-23 sulfated sterols from a marine source endowed with a PXR agonistic activity. J. Med. Chem..

[9] Sepe V., Ummarino R., D’Auria M.V., Mencarelli A., D’Amore C., Renga B., Zampella A., Fiorucci S. (2011). Total synthesis and pharmacological characterization of solomonsterol A, a potent marine pregnane-X-receptor agonist endowed with anti-inflammatory activity. J. Med. Chem..

[10] Sepe V., Ummarino R., D’Auria M.V., Renga B., Fiorucci S., Zampella A. (2012). The first total synthesis of solomonsterol B, a marine pregnane X receptor agonist. Eur. J. Org. Chem..

[11] Sepe V., Ummarino R., D’Auria M.V., Lauro G., Bifulco G., D’Amore C., Renga B., Fiorucci S., Zampella A. (2012). Modification in the side chain of solomonsterol A: Discovery of cholestan disulfate as a potent pregnane-X-receptor agonist. Org. Biomol. Chem..

[12] De Marino S., Festa C., D’Auria M.V., Cresteil T., Debitus C., Zampella A. (2011). Swinholide J, a potent cytotoxin from the marine sponge *Theonella swinhoei*. Mar. Drugs.

[13] De Marino S., Ummarino R., D’Auria M.V., Chini M.G., Bifulco G., Renga B., D’Amore C., Fiorucci S., Debitus C., Zampella A. (2011). Theonellasterols and conicasterols from *Theonella swinhoei*. Novel marine natural ligands for human nuclear receptors. J. Med. Chem..

[14] De Marino S., Sepe V., D’Auria M.V., Bifulco G., Renga B., Petek S., Fiorucci S., Zampella A. (2011). Towards new ligands of nuclear receptors. Discovery of malaitasterol A, an unique bis-secosterol from marine sponge *Theonella swinhoei*. Org. Biomol. Chem..

[15] Sepe V., Ummarino R., D’Auria M.V., Chini M.G., Bifulco G., Renga B., D’Amore C., Debitus C., Fiorucci S., Zampella A. (2012). Conicasterol E, a small heterodimer partner sparing farnesoid X receptor modulator endowed with a pregnane X receptor agonistic activity, from the marine sponge *Theonella swinhoei*. J. Med. Chem..

[16] Chini M.G., Jones C.R., Zampella A., D’Auria M.V., Renga B., Fiorucci S., Butts C.P., Bifulco G. (2012). Quantitative NMR-derived interproton distances combined with quantum mechanical calculations of 13C chemical shifts in the stereochemical determination of conicasterol F, a nuclear receptor ligand from *Theonella swinhoei*. J. Org. Chem..

[17] De Marino S., Ummarino R., D’Auria M.V., Chini M.G., Bifulco G., D’Amore C., Renga B., Mencarelli A., Petek S., Fiorucci S., Zampella A. (2012). 4-Methylenesterols from *Theonella swinhoei* sponge are natural pregnane-X-receptor agonists and farnesoid-X-receptor antagonists that modulate innate immunity. Steroids.

[18] Fiorucci S., Distrutti E., Bifulco G., D’Auria M.V., Zampella A. (2012). Marine sponge steroids as nuclear receptor ligands. Trends Pharmacol. Sci..

[19] Pellicciari R., Costantino G., Fiorucci S. (2005). Farnesoid X receptor: From structure to potential clinical applications. J. Med. Chem..

[20] Fiorucci S., Rizzo G., Donini A., Distrutti E., Santucci L. (2007). Targeting farnesoid X receptor for liver and metabolic disorders. Trends Mol. Med..

[21] Sonoda J., Xie W., Rosenfeld J.M., Barwick J.L., Guzelian P.S., Evans R.M. (2002). Regulation of a xenobiotic sulfonation cascade by nuclear pregnane X receptor (PXR). Proc. Natl. Acad. Sci. USA.

[22] Guo G.L., Lambert G., Negishi M., Ward J.M., Brewer H.B.J., Kliewer S.A., Gonzalez F.J., Sinal C.J. (2003). Complementary roles of farnesoid X receptor, pregnane X receptor, and constitutive androstane receptor in protection against bile acid toxicity. J. Biol. Chem..

[23] Fiorucci S., Cipriani S., Mencarelli A., Baldelli F., Bifulco G., Zampella A. (2011). Farnesoid X receptor agonist for the treatment of liver and metabolic disorders: Focus on 6-ethyl-CDCA. Mini Rev. Med. Chem..

[24] Kakizaki S., Takizawa D., Tojima H., Horiguchi N., Yamazaki Y., Mori M. (2011). Nuclear receptors CAR and PXR; therapeutic targets for cholestatic liver disease. Front Biosci..

[25] Fiorucci S., Zampella A., Distrutti E. (2012). Development of FXR, PXR and CAR agonists and antagonists for treatment of liver disorders. Curr. Top. Med. Chem..

[26] Poupon R.E., Bonnand A.M., Chrétien Y., Poupon R. (1999). Ten-Year survival in ursodeoxycholic acid-treated patients with primary biliary cirrhosis. Hepatology.

[27] Parés A., Caballería L., Rodés J. (2006). Excellent long-term survival in patients with primary biliary cirrhosis and biochemical response to ursodeoxycholic acid. Gastroenterology.

[28] Renga B., Mencarelli A., D’Amore C., Cipriani S., D’Auria M.V., Sepe V., Chini M.G., Monti M.C., Bifulco G., Zampella A., Fiorucci S. (2012). Discovery that theonellasterol a marine sponge sterol is a highly selective FXR antagonist that protects against liver injury in cholestasis. PLoS One.

[29] Sepe V., Bifulco G., Renga B., D’Amore C., Fiorucci S., Zampella A. (2011). Discovery of sulfated sterols from marine invertebrates as a new class of marine natural antagonists of farnesoid-X-receptor. J. Med. Chem..

[30] D’Auria M.V., Sepe V., Zampella A. (2012). Natural ligands for nuclear receptors: Biology and potential therapeutic applications. Curr. Top. Med. Chem..

[31] Pellicciari R., Gioiello A., Costantino G., Sadeghpour B.M., Rizzo G., Meyer U., Parks D.J., Entrena-Guadix A., Fiorucci S. (2006). Back door modulation of the farnesoid X receptor: Design, synthesis, and biological evaluation of a series of side chain modified chenodeoxycholic acid derivatives. J. Med. Chem..

[32] Mi L.Z., Devarakonda S., Harp J.M., Han Q., Pellicciari R., Willson T.M., Khorasanizadeh S., Rastinejad F. (2003). Structural basis for bile acid binding and activation of the nuclear receptor FXR. Mol. Cell.

[33] Schmidt A.W., Doert T., Goutal S., Gruner M., Mende F., Kurzchalia T.V., Knölker H.J. (2006). Regio- and stereospecific synthesis of cholesterol derivatives and their hormonal activity in *Caenorhabditis elegans*. Eur. J. Org. Chem..

[34] Morris G.M., Huey R., Lindstrom W., Sanner M.F., Belew R.K., Goodsell D.S., Olson A.J. (2009). AutoDock4 and AutoDockTools4: Automated docking with selective receptor flexibility. J. Comput. Chem..

[35] Sanner M.F. (1999). A programming language for software integration and development. J. Mol. Graphics.

